# Fe(III) Complexes Based on Mono*-* and Bis-pyrazolyl-*s*-triazine Ligands: Synthesis, Molecular Structure, Hirshfeld, and Antimicrobial Evaluations

**DOI:** 10.3390/molecules25235750

**Published:** 2020-12-05

**Authors:** Saied M. Soliman, Hessa H. Al-Rasheed, Jörg H. Albering, Ayman El-Faham

**Affiliations:** 1 Department of Chemistry, Faculty of Science, Alexandria University, P.O. Box 426, Ibrahimia, Alexandria 21321, Egypt; 2 Department of Chemistry, College of Science, King Saud University, P.O. Box 2455, Riyadh 11451, Saudi Arabia; halbahli@ksu.edu.sa; 3 Graz University of Technology, Mandellstr. 11/III, A-8010 Graz, Austria; joerg.albering@tugraz.at

**Keywords:** pyrazolyl-*s*-triazine, Fe(III), self-assembly, Hirshfeld, antimicrobial activity

## Abstract

The self-assembly of iron(III) chloride with three pyrazolyl-*s*-triazine ligands, namely 2,4-bis(3,5-dimethyl-1*H*-pyrazol-1-yl)-6-(piperidin-1-yl)-1,3,5-triazine (^Pip^**BPT**), 4-(4,6-bis(3,5-dimethyl-1*H*-pyrazol-1-yl)-1,3,5-triazin-2-yl)morpholine (^Morph^**BPT**), and 4,4’-(6-(3,5-dimethyl-1*H*-pyrazol-1-yl)-1,3,5-triazine-2,4-diyl)dimorpholine (^bisMorph^**PT**) afforded [Fe(^Pip^BPT)Cl_2_][FeCl_4_] (**1**), [Fe(^Morph^BPT)Cl_2_][FeCl_4_] (**2**), and [H(^bisMorph^PT)][FeCl_4_]. ^bisMorph^PT.2H_2_O (**3**), respectively, in good yield. In complexes **1** and **2**, the Fe(III) is pentacoordinated with three Fe-N interactions from the pincer ligand and two coordinated chloride anions in the inner sphere, and FeCl_4_¯ in the outer sphere. Complex **3** is comprised of one protonated ligand as cationic part, one FeCl_4_¯ anion, and one neutral ^bisMorph^**PT** molecule in addition to two crystallized water molecules. Analysis of molecular packing using Hirshfeld calculations indicated that H…H and Cl…H are the most important in the molecular packing. They comprised 40.1% and 37.4%, respectively in **1** and 32.4% and 37.8%, respectively in **2**. Complex **1** exhibited the most bioactivity against the tested microbes while **3** had the lowest bioactivity. The ^bisMorph^**PT** and ^Morph^**BPT** were inactive towards the tested microbes while ^Pip^**BPT** was active. As a whole, the Fe(III) complexes have enhanced antibacterial and antifungal activities as compared to the free ligands.

## 1. Introduction

Iron is a readily available element, as it is considered to be one of the most abundant. It is cheap and has almost-negligible hazardous effects on the environment as it has low toxicity [1,2,3]. Iron compounds play a crucial role in ammonia production by the Haber–Bosch process. On other hand, iron and its compounds have a key role in homogenous molecular catalysis [4,5,6,7].

Bis-pyrazolyl-*s*-triazine (**BPT**) ligands are a class of chelators which have been utilized in the synthesis of several divalent metal ion complexes with interesting molecular and supramolecular structures [8,9,10,11,12,13]. These *s*-triazine pincer-type complexes can be easily synthesized using self-assembly in a water-alcohol mixture. Additionally, they have extra-stability due to the chelate effect. Although iron has low toxicity, there are many problems due to high iron overload because it plays a major role in the generation of free radicals [14,15]. **BPT** ligands have key characteristics to act as a solution for this problem because they are powerful chelators. 

On other hand, several organic-based antibacterial and antifungal drugs were discovered over the last few years [16]. Many of these antibiotics cannot overcome the problem of multidrug-resistant microbes [17,18,19]. Therefore, the replacement of these traditional antibiotics by other medications that can solve the problem of antibiotic-resistant pathogens has become an urgent need [17,18,19]. In this regard, some Fe(III) complexes have good antibacterial activity against a broad range of bacteria, but not fungi [20]. Others were found to have good antibacterial and moderate antifungal activities [20].

**Figure 1:**
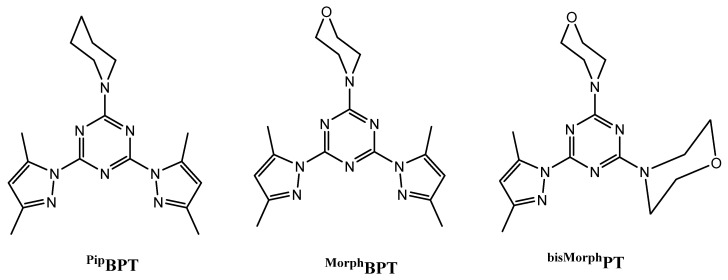
Structure of the *mono*- and bis-pyrazolyl-*s*-triazine ligands [21,22]. Ligands shown are: 2,4-bis(3,5-dimethyl-1*H*-pyrazol-1-yl)-6-(piperidin-1-yl)-1,3,5-triazine (^Pip^**BPT**), 4-(4,6-bis(3,5-dimethyl-1*H*-pyrazol-1-yl)-1,3,5-triazin-2-yl)morpholine (^Morph^**BPT**), and 4,4’-(6-(3,5-dimethyl-1*H*-pyrazol-1-yl)-1,3,5-triazine-2,4-diyl)dimorpholine (^bisMorph^**PT**).

In the present work, we self-assembled three Fe(III) complexes by the direct reaction of FeCl_3_ with the mono*-* and bis-pyrazolyl-*s*-triazine ligands shown in Figure 1. Their structure aspects were studied using single-crystal X-ray diffraction in combination with Hirshfeld analysis. The antibacterial and antifungal activities of these Fe(III) complexes are also presented. 

## 2. Results and Discussion

### 2.1. Structure Description

The crystals of the synthesized complexes were simply obtained from the direct reaction of the Fe(III) salt with the functional ligand in water-ethanol solvent mixture at room temperature using self-assembly. The X-ray single-crystal structure of the Fe(III) complexes are presented for the first time and the crystal data are listed in Table 1.

#### 2.1.1. Crystal Structure Description of [Fe(^Pip^**BPT**)Cl_2_][FeCl_4_] (**1**)

**Figure 2:**
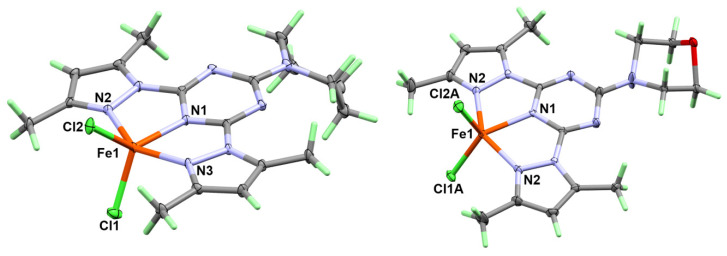
X-ray structure of complexes **1** (left) and **2** (right), the atoms have been drawn at a 30% probability level. The [FeCl_4_]^-^ anions in the outer sphere were omitted for better clarity.

Complex **1** crystallizes in the monoclinic crystal system with the space group P2_1_/c and Z = 4; the asymmetric unit comprises one [Fe(^Pip^**BPT**)Cl_2_][FeCl_4_] unit. The structure of the inner sphere complex in **1** consists of one cationic complex unit in which the Fe(III) ion is coordinated by ^Pip^**BPT** in a tridentate pincer fashion and two chloride ions. The outer sphere is an anion: a tetrahedral FeCl_4_¯ unit (Figure 2). The Fe-N distances are significantly shorter for the Fe-N(*s*-triazine) than the Fe-N(pyrazole), where the two Fe-N(pyrazole) bonds are only slightly different (Table 2). The two Fe1-Cl1 and Fe1-Cl2 bonds have very close bond distances of 2.1699(6) Å and 2.1766(6) Å, respectively. The coordination geometry of the five-coordinated Fe(III) ion is described using Addison criteria [23]. The coordination geometry, as shown in Figure 2, lies between the square pyramid and the trigonal bipyramid with a N3-Fe1-N2 angle (β) of 146.46(6)° and N1-Fe1-Cl2 angle (α) of 133.34(5)°, giving a τ = ((β − α)/60) value of 0.22. As a result, the coordination geometry around Fe(III) could be described as a distorted square pyramid.

**Figure 3:**
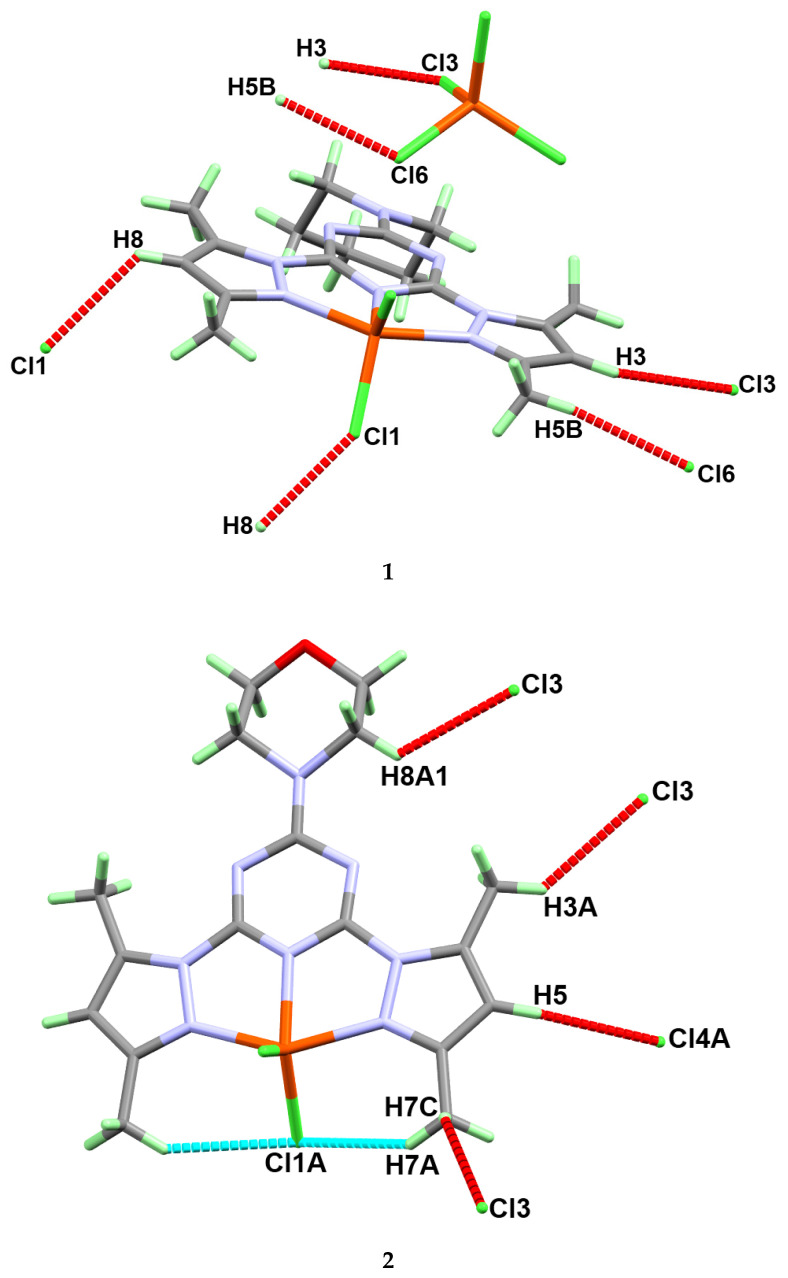
The hydrogen bond contacts in complexes **1** (upper) and **2** (lower).

**Figure 4:**
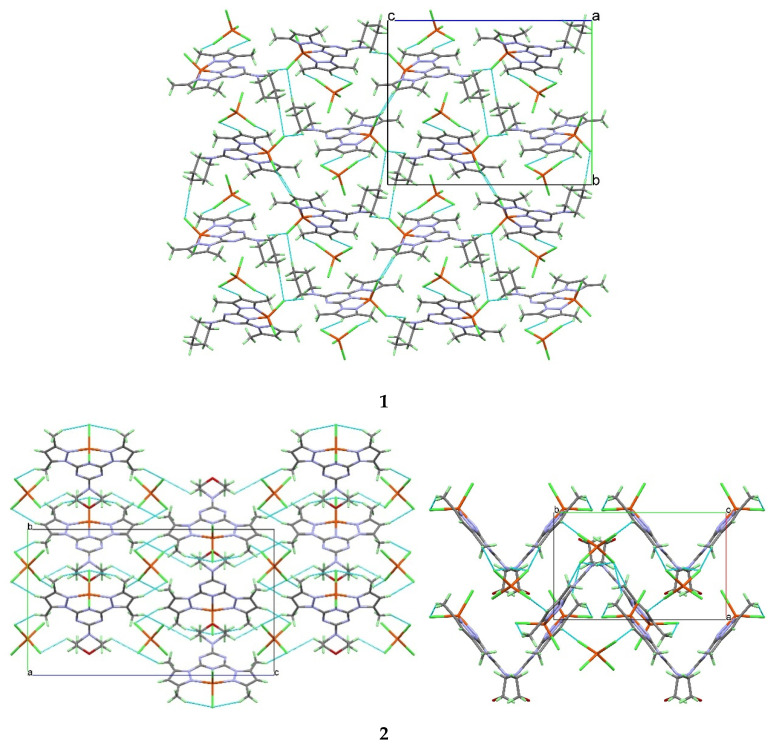
The hydrogen bond polymers in the crystal structures of complexes **1** (upper, view along the *a*-axis) and **2** (lower, views along the *a*- and *c*-axes). The hydrogen bridges are shown as light-blue dotted lines.

The molecules of **1** are packed mainly by Cl…H hydrogen bonds as shown in Figure 3 (upper part) and listed in Table 3. The donor–acceptor distances are 3.428(2) Å, 3.622(2) Å, and 3.723(2) Å for C8-H8…Cl1, C3-H3…Cl3, and C5-H5B…Cl6 hydrogen bonding interactions, respectively. The packing of complex molecules is shown in Figure 4 (upper part). The network connected via Cl…H bridge interactions shows a 3D connectivity.

#### 2.1.2. Crystal Structure Description of [Fe(^Morph^BPT)Cl_2_][FeCl_4_] (**2**)

Complex **2** has a very close structure to **1**, with one [Fe(^Morph^**BPT**)Cl_2_]^+^ as an inner sphere complex and [FeCl_4_]¯ as a counter ion. The major difference is that complex **2** crystallizes in the more symmetric orthorhombic crystal system and space group Pbcm with half molecular formula as asymmetric unit. The molecule comprises a symmetrical plane passing vertically through the molecule, intersecting the Fe(III) center and the two chloride anions, and splitting the organic ligand into two halves. In this regard, there are two equidistant Fe-N(pyrazole) bonds with iron to nitrogen distance of 2.099(2) Å and one shorter Fe-N(*s*-triazine) bond (2.036(3) Å). List of the most important bond distances are given in Table 4. The coordination sphere is completed by the two coordinated chloride anions with iron to chlorine distances ranging from 2.090(6)–2.262(5) Å for the two disordered parts (Figure S4, Supplementary Materials). The Addison criteria τ for the two complex parts are 0.26 and 0.05 for the disordered parts A and B, respectively. These calculations indicate that the two complex parts have different coordination geometries: part **B** is closer to being a more perfect square pyramid than part **A**. Regarding the scale factors for the two domains A and B, both are close 0.5. Thus, if the Cl1A and Cl2B atoms, as well as Cl1B and Cl2A, are assumed to belong to the same polyhedron, the τ values are calculated to be same (τ = 0.26 and 0.05), which confirms our conclusion.

The different hydrogen bridge contacts controlling the molecular packing of complex **2** are listed in Table 3 and shown in Figure 3, while the molecular packing showing the different molecular units packed via C-H…Cl interactions is shown in the lower part of Figure 4. Complex **2** also shows a 3D network connected via Cl…H interactions. Although the coordination modes of both complexes **1** and **2** look very similar, the hydrogen bridge networks turn out to be quite different. Small changes of the ligand molecule lead to significant differences in the packing.

#### 2.1.3. Crystal Structure Description of [H(^bisMorph^**PT**)][FeCl_4_] ^bisMorph^**PT**.2H_2_O (**3**)

Attempts to synthesize a coordination complex compound of the ^bisMorph^**PT** ligand with FeCl_3_ have failed so far. The only crystalline compound that was found was a **[H(**^bisMorph^**PT)][FeCl_4_**] salt with one co-crystallized ^bisMorph^**PT** ligand and two crystal water molecules in the asymmetric unit (Z = 2) of the triclinic unit cell with the symmetry P-1.

**Figure 5:**
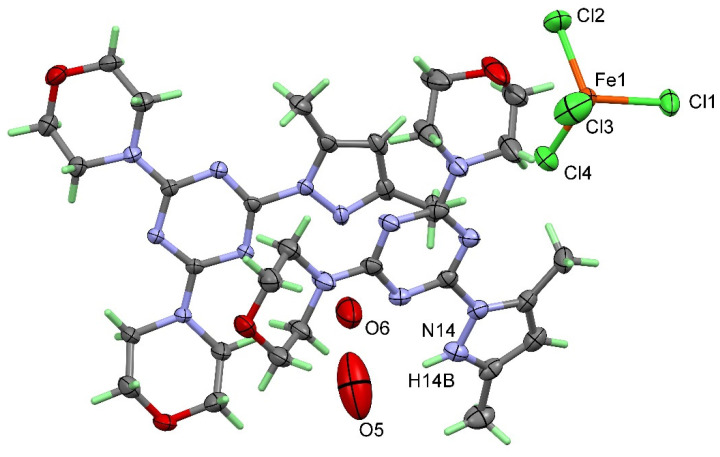
X-ray structure of compound **3**. All atoms have been drawn at a 50% probability level.

Compound **3** comprises four parts: the protonated organic ligand **[H(**^bisMorph^**PT)]^+^** as a cationic part, one electrically neutral ^bisMorph^**PT** molecule, a negatively charged [FeCl_4_]¯ ion, and two crystal water molecules in a void of the packing (Figure 5). The crystal quality of this compound was not very good. We found some disorder in the organic part of the crystal structure, and the protons of the crystal water molecules were not detectable. For these reasons, we only give the crystallographic data in this publication and do not further describe its molecular and supramolecular aspects in detail. Although our attempts to synthesize a complex containing the ^bisMorph^**PT** ligand and FeCl_3_ were not successful, it does not necessarily mean that such a compound does not exist. In any case, it seemed to be useful to publish the data of compound **3** found in this context in order to create a reference for subsequent work. One possible reason for not obtaining a coordination complex of Fe(III) with the bidentate ^bisMorph^PT ligand is its lower denticity compared to the tridentate ^Pip^BPT and ^Morph^BPT pincer chelates. Another possible reason is the steric effect resulting from the replacement of one pyrazole moiety by the morpholine one. The latter has no coordinating ability and a more bulky character that prevents the ^bisMorph^**PT** from coordinating to the Fe(III) ion.

### 2.2. Analysis of Molecular Packing

**Figure 6:**
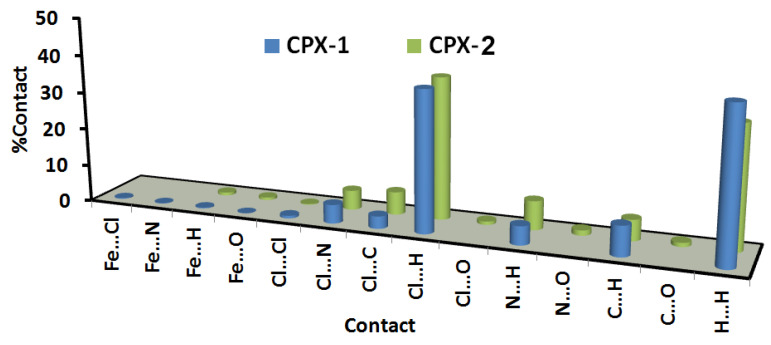
All intermolecular interactions in complexes (CPXs) **1** and **2**.

**Figure 7:**
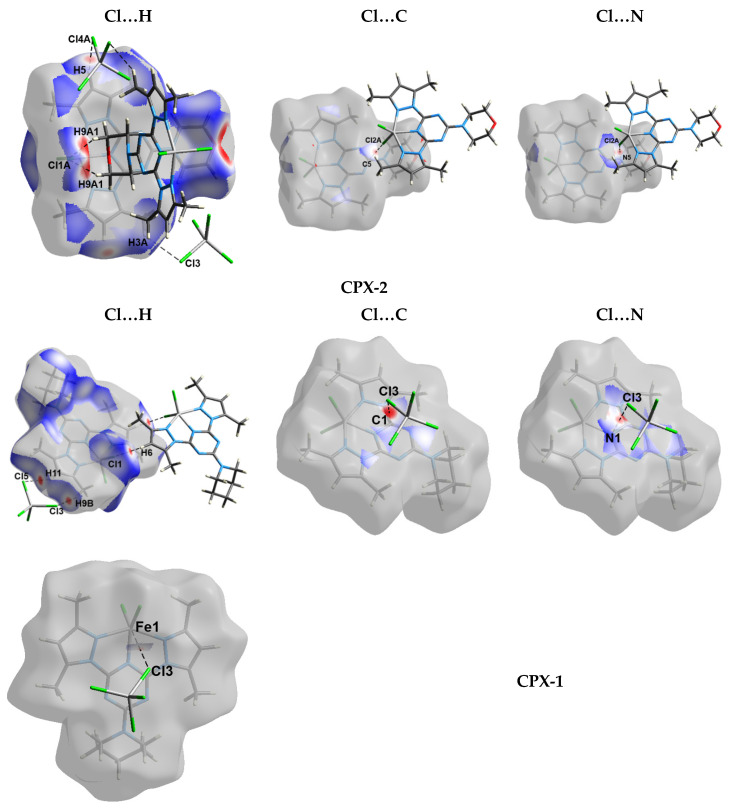
The decomposed d_norm_ maps and fingerprint plots of the most important contacts in **1** and **2**.

Hirshfeld surfaces mapped over d_norm_, shape index (SI), and curvedness for complexes **1** and **2** are shown in Figure S5 (Supplementary Materials). A summary of the most important contacts and their percentages are shown in Figure 6, while the decomposed d_norm_ maps of the short and most significant contacts in the studied complexes are collected in Figure 7. The decomposed fingerprint plots indicate the same common contacts in both complexes, which are H…H and Cl…H interactions, the most abundant intermolecular interactions in the studied complexes. The percentages of these contacts are 40.1% and 37.4% in complex **1**, respectively while they are 32.4% and 37.8% in complex **2**, respectively. The Cl…H hydrogen bonds appear as red regions in the Hirshfeld d_norm_ maps in both complexes and indicate shorter contact distances than the van der Waals (vdW) radii sum of H and Cl atoms. The anion (FeCl_4_¯)-π stacking interactions are significant in both complexes. Complexes **1** and **2** show significantly short C…Cl and N…Cl contacts, with interaction distances also found to be shorter than the van der Waals radii sum of the two elements sharing this contact (Figure 7). The contact distances of the N…Cl interactions are 3.213 Å and 3.226 Å for complexes **1** and **2**, respectively, while the C…Cl contact distances are 3.257 Å and 3.381 Å for complexes **1** and **2**, respectively. In complex **1**, there is one short Fe1…Cl3 interaction (3.708 Å) between the complex cation and one of the Cl atoms from the complex anion (FeCl_4_¯).

### 2.3. Antimicrobial Activity of the Studied Compounds

#### 2.3.1. Inhibition Zones

In the current study, the antibacterial activity of the free ligands as well as compounds **1**–**3** were tested against Gram-positive bacteria, namely, *Staphylococcus aureus* (ATCC 29213) and *Staphylococcus epidermidis* (ATCC 12228); and Gram-negative bacteria, namely, *Escherichia coli* (ATCC 25922) and *Pseudomonas aeruginosa* (ATCC 27853) [24,25,26,27]. The free ^Morph^**BPT** and ^bisMorph^**PT** ligands were inactive against the target microbes at the applicable concentration. On other hand, **^Pip^BPT** showed good activity against *S. aureus*, *S. epidermidis, P. aeruginosa*, and *Candida albicans (*ATCC 60193) and it was completely inactive against *E. coli* (Table 5). In contrast, Fe(III) compounds **1**–**3** showed more potent activities against the target pathogenic microbes than did the corresponding free ligands, as illustrated from the values of the inhibition zones (mm) in Table 5. The values are considered an indicator for the bioactivity of the tested compounds at a concentration of 200 µg/mL. Compounds **1**–**3** appeared to have a more potent bioactivity against the target Gram-positive pathogenic bacteria than against the Gram-negative ones, and showed potent activity against the tested fungus (*C. albicans*).

Complex **1** showed the most potency as an antibacterial and antifungal agent against all the target microbes, while **3** showed the lowest bioactivity. Additionally, complex **1** (18 mm) had better antifungal activity than the standard fluconazole (14 mm). Complex **1** had better antibacterial action against *S. epidermidis* (23 mm) and *P. aeruginosa* (22 mm) than the standard drug gentamycin (22 mm and 19 mm, respectively).

The antimicrobial activities of the studied compounds were also evaluated at different concentrations of 100 µg/mL, 200 µg/mL, and 300 µg/mL per disc, as shown in Table 6. Compounds **1**–**3** at these concentrations showed moderate to strong activity against all tested microbes, even at the lowest concentration of 100 μg/mL, where the best results were obtained for complex **1**. On the other hand, as the concentration of the tested compound increased, the inhibition zone also increased. This result reveals that the presence of the piperidine/bis*-*pyrazolo combination with Fe(III) in one compound is the key for the bioactivity. These data agree with the literature, where the presence of piperidine enhanced the activity compared to the analogous morpholine derivatives [27].

#### 2.3.2. Minimum Inhibitory Concentration (MIC) and Minimum Bactericidal Concentration (MBC)

The minimum inhibitory concentrations (MIC) of compounds **1**–**3** are given in Table 7. All tested compounds were active against *S. aureus*, *S. epidermidis*, *E. coli*, *P. aeruginosa*, and *C. albicans*. Again, complex **1** had the lowest MIC and MBC values, indicating its higher potency against all tested microbes as compared to **2** and **3** (Table 7).

## 3. Experimental

### 3.1. Materials and Physical Measurements

Chemicals were purchased from Sigma-Aldrich Company. CHN analyses were performed using a Perkin Elmer 2400 Elemental Analyzer.

### 3.2. Syntheses

#### 3.2.1. Synthesis of *s*-Triazine-Based Ligands

The ligands ^Pip^BPT, ^Morph^BPT, and ^bisMorph^PT were prepared following the method reported by our research group [21,22] (Supplementary Materials, Method S1 and Method S2, Figures S1–S3).

#### 3.2.2. Synthesis of Fe(III) Complexes

All of the studied complexes were synthesized using a self-assembly technique, by mixing the aqueous solution of FeCl_3_ (1 mmol, 162 mg) with the ethanolic solution of the functional ligand. The resulting clear solutions were left for slow evaporation until plate-like brown crystals of the target complexes were formed. The resulting crystals were collected by filtration and were found suitable for single-crystal X-ray diffraction measurements.

Yield: C_18_H_24_N_8_Fe_2_Cl_6_ (**1**) 73% with respect to the ligand. Anal. Calc. C, 31.94; H, 3.57; N, 16.56%. Found: C, 31.80; H, 3.51; N, 16.43%.

Yield: C_17_H_22_N_8_OFe_2_Cl_6_ (**2**) 76% with respect to the ligand. Anal. Calc. C, 30.08; H, 3.27; N, 16.51%. Found: C, 29.90; H, 3.21; N, 16.38%.

Yield: C_32_H_51_N_14_O_6_ Fe Cl_4_ (**3**) 70% with respect to the ligand. Anal. Calc. C, 41.53; H, 5.55; N, 21.19%. Found: C, 41.35; H, 5.49; N, 21.01%.

### 3.3. Crystal Structure Determination

The crystal structures of complexes **1**–**3** were determined using a Bruker D8 Quest diffractometer employing SHELXTL and SADABS programs [28,29,30]. Hirshfeld calculations were performed using the default parameters of the Crystal Explorer 17.5 program [31,32,33,34,35].

### 3.4. Antimicrobial Studies

We determined the antimicrobial activities of the free ligands, as well as those of the corresponding Fe(III) complexes, against different microbes [22]. More details regarding the antimicrobial assay are found in Supplementary Materials (Methods S3–S6).

## 4. Conclusions

Three self-assembled Fe(III) complexes were synthesized by direct reaction of iron(III) chloride and the functional ligand in a water-alcohol medium. All complexes were obtained in good yield and their structures were confirmed by single-crystal X-ray diffraction. The supramolecular structures of complexes **1** and **2** were analyzed using Hirshfeld calculations with the aid of the CIF data. The Fe(III) complexes were bioactive against the target microbes and generally more active than the functional ligands. It was found that the combination of piperidine and bispyrazolo moieties with Fe(III) in one compound (**1**) had the best bioactivity in comparison with the corresponding complexes (**2** and **3**) comprised of morpholine group(s).

## Figures and Tables

**Table 1:** Crystal data and structure refinement for the studied complexes.

Compound	1	2	3
Empirical formula	C_18_H_24_Cl_6_Fe_2_N_8_	C_17_H_22_Cl_6_Fe_2_N_8_O	C_32_H_51_Cl_4_FeN_14_O_6_
Formula weight (g/mol)	676.85	678.82	925.51
Temperature (K)	119(2)	124(2)	293(2)
λ (Å)	0.71073	0.71073	0.71073
Crystal system	Monoclinic	Orthorhombic	Triclinic
Space group	P2_1_/c	Pbcm	P-1
Unit cell dimensions			
a (Å)	8.9549(3)	8.5201(3)	12.4352(15)
b (Å)	15.7871(6)	13.7094(5)	12.8632(16)
c (Å)	19.9063(7)	23.2383(9)	15.6509(19)
α (°)	90	90	76.955(3)
β (°)	99.457(2)	90	89.531(3)
γ (°)	90	90	66.926(3)
Volume (Å^3^)	2775.9(2)	2714.4(2)	2234.7(5)
Z	4	4	2
Density (calculated, g/cm^3^)	1.620	1.661	1.370
Absorption coefficient (mm^−1^)	1.647	1.687	0.633
F(000)	1368	1368	958
Crystal size (mm^3^)	0.29 × 0.16 × 0.09	0.04 × 0.12 × 0.15	0.26 × 0.20 × 0.08
θ range (°)	2.31 to 25.49	2.81 to 24.99	2.33 to 25.09
Index ranges	−10 ≤ h ≤ 10, −19 ≤ k ≤ 19, −24 ≤ l ≤ 24	−10 ≤ h ≤ 10, −16 ≤ k ≤ 16, −27 ≤ l ≤ 27	−14 ≤ h ≤ 14, −15 ≤ k ≤ 15, −18 ≤ l ≤ 18
Reflections collected	38,064	21,560	64,462
Independent reflections	5139 [R(int) = 0.0427]	2453 [R(int) = 0.0294]	7915 [R(int) = 0.0769]
Completeness to theta (%)	99.8	99.90	99.5
Refinement method	Full-matrix least-squares on F^2^		
Data/restraints/parameters	5139/0/311	2453/0/207	7909/0/528
Goodness-of-fit on F^2^	1.045	1.083	1.008
Final R indices [I > 2sigma(I)]	R1 = 0.0238, wR2 = 0.0530	R1 = 0.0397, wR2 = 0.0988	R_1_ = 0.0932, wR_2_ = 0.2007
R indices (all data)	R1 = 0.0334, wR2 = 0.0571	R1 = 0.0440, wR2 = 0.1024	R_1_ = 0.1654, wR_2_ = 0.2464
Largest diff. peak and hole	0.291 and −0.306	0.621 and −1.035	0.87and −0.64
CCDC No.	2044018	2044016	2044017

**Table 2:** Bond distances and angles in **1**.

**Atoms**	**Distance (Å)**	**Atoms**	**Distance (Å)**
Fe1-N1	2.0295(15)	Fe2-Cl3	2.1840(6)
Fe1-N3	2.0940(16)	Fe2-Cl4	2.1836(6)
Fe1-N2	2.1092(16)	Fe2-Cl5	2.1791(6)
Fe1-Cl1	2.1699(6)	Fe2-Cl6	2.1873(6)
Fe1-Cl2	2.1766(6)		
**Atoms**	**Angle (°)**	**Atoms**	**Angle (°)**
N1-Fe1-N3	73.24(6)	N2-Fe1-Cl1	100.76(5)
N1-Fe1-N2	73.65(6)	N1-Fe1-Cl2	133.34(5)
N3-Fe1-N2	146.46(6)	N3-Fe1-Cl2	99.11(5)
N1-Fe1-Cl1	117.06(5)	N2-Fe1-Cl2	99.58(5)
N3-Fe1-Cl1	98.84(5)	Cl1-Fe1-Cl2	109.58(3)

**Table 3:** Hydrogen bond parameters of complexes **1** and **2**.

Atoms	D-H (Å)	H…A (Å)	D…A (Å)	D-H…A (°)
**Complex 1**				
C3-H3…Cl3 ^i^	0.95	2.77	3.622(2)	149
C5-H5B…Cl6 ^i^	0.98	2.8	3.723(2)	158
C8-H8…Cl1^ii^	0.95	2.78	3.428(2)	126
^i^ 1 + x,y,z ^i^ 1 + x,y,z ^ii^ 1-x,-y,1-z and				
**Complex 2**				
C5-H5…Cl4A ^i^	0.95	2.79	3.651(5)	152
C9A-H9A1…Cl1A ^ii^	0.99	2.55	2.916(7)	101
^i^ x,-1 + y,z and ^ii^ 1-x,1/2 + y,3/2-z				

**Table 4:** Bond distances and angles in **2**.

**Atoms**	**Distance (Å)**	**Atoms**	**Distance (Å)**
Fe1-N1	2.038(3)	Fe2-Cl4B	2.085(3)
Fe1-N2 ^1^	2.099(3)	Fe2-Cl3 ^2^	2.1667(10)
Fe1-N2	2.099(3)	Fe2-Cl3	2.1668(10)
Fe1-Cl1A	2.250(3)	Fe2-Cl4A	2.329(3)
Fe1-Cl2A	2.262(5)		
Fe1-Cl1B	2.151(2)		
Fe1-Cl2B	2.090(6)		
**Atoms**	**Angle (°)**	**Atoms**	**Angle (°)**
N1-Fe1-N2 ^1^	73.66(7)	Cl2B-Fe1-N2	101.82(8)
N1-Fe1-N2	73.66(7)	N1-Fe1-Cl1B	149.41(12)
N2 ^1^-Fe1-N2	146.19(13)	Cl2B-Fe1-Cl1B	90.49(17)
N1-Fe1-Cl1A	130.50(12)	N2 ^1^-Fe1-Cl1B	101.81(7)
N2^1^-Fe1-Cl1A	97.28(7)	N2-Fe1-Cl1B	101.81(7)
N2-Fe1-Cl1A	97.28(7)	Cl1A-Fe1-Cl2A	122.17(15)
N1-Fe1-Cl2A	107.33(16)	Cl4B-Fe2-Cl3 ^2^	106.87(8)
N2 ^1^-Fe1-Cl2A	98.86(8)	Cl4B-Fe2-Cl3	120.70(11)
N2-Fe1-Cl2A	98.86(8)	Cl3^2^-Fe2-Cl3	109.97(6)
N1-Fe1-Cl2B	120.10(17)	Cl3^2^-Fe2-Cl4A	105.43(8)
Cl2B-Fe1-N2 ^1^	101.82(8)	Cl3-Fe2-Cl4A	102.31(10)

^1^ X,Y,3/2-Z and ^2^ +X,3/2-Y,1-Z.

**Table 5:** Anti-microbiological activities of the studied compounds against some tested microbes at 200 µg by the agar well diffusion method.

Test Compounds	Microbes				
	*Staphylococcus aureus*	*Staphylococcus epidermidis*	*Escherichia coli*	*Pseudomonas aeruginosa*	*Candida albicans*
^Pip^ **BPT**	11	17	-	13	12
^Morph^ **BPT**	-	-	-	-	-
^bisMorph^ **PT**	-	-	-	-	-
**1**	25	23	19	22	18
**2**	18	19	16	17	14
**3**	17	16	14	15	12
**Fluconazole**	-	-	-	-	14
**Gentamycin**	28	22	21	19	-

**Table 6:** Antimicrobial activities of ^Pip^**BPT** and **1**–**3** at different concentrations.

Compounds	Organism	Concentration		
		100	200	300
**1**	*E. coli*	16	19	21
	*P. aeruginosa*	20	22	23
	*S. aureus*	19	25	26
	*S. epidermidis*	18	23	25
	*C. albicans*	14	18	20
^Pip^ **BPT**	*E. coli*	-	-	-
	*P. aeruginosa*	13	15	18
	*S. aureus*	17	18	19
	*S. epidermidis*	11	13	15
	*C. albicans*	10	11	13
**2**	*E. coli*	14	16	18
	*P. aeruginosa*	14	17	18
	*S. aureus*	14	18	19
	*S. epidermidis*	15	19	20
	*C. albicans*	12	14	16
**3**	*E. coli*	12	15	17
	*P. aeruginosa*	12	15	17
	*S. aureus*	14	17	19
	*S. epidermidis*	13	16	17
	*C. albicans*	11	12	15

**Table 7:** Minimum inhibitory concentrations (MIC) (µg/mL) and minimum bactericidal concentrations (MBC) (µg/mL) of **1**–**3** against the growth of target microbes.

Microbes	[Fe(^Pip^BPT)Cl_2_][FeCl_4_] (1)		[Fe(^Morph^BPT)Cl_2_][FeCl_4_] (2)		[H(^bisMorph^PT)][FeCl_4_]. ^bisMorph^PT (3)	
	MIC	MBC	MIC	MBC	MIC	MBC
*S. epidermidis*	8.3	16.6	9.7	19.4	18.8	37.5
*S. aureus*	8.7	17.5	9.8	19.6	18.8	37.5
*E. coli*	8.7	17.5	9.8	19.6	18.8	37.5
*P. aeruginosa*	8.2	16.5	9.8	19.6	18.8	37.5
*C. albicans*	18.8	100.0	37.5	150.0	37.5	150.0

## Supplementary Materials

The following are available online. Figure S1: ^1^H-NMR and ^13^C-NMR of *^Morph^***BPT***,* Figure S2: ^1^H-NMR and ^13^C-NMR of *^Pip^***BPT**, Figure S3: ^1^H- and ^13^C-NMR for *^bisMorph^***PT**, Figure S4: Structure showing the disordered parts in **2**. Figure S5: Hirshfeld surfaces mapped over d_norm_, shape index, and curvedness. In addition, the detailed synthesis of the ligands and biological experiments are given in the Supplementary data file. Methods S1–S6.
