# Supplementary material for: Fe(III) Complexes Based on Mono- and Bis-pyrazolyl-s-triazine Ligands: Synthesis, Molecular Structure, Hirshfeld, and Antimicrobial Evaluations
Source: Molecules. 2020 Dec 5;25(23):5750. doi: 10.3390/molecules25235750 (PMC7730701; doi:10.3390/molecules25235750)
Supplement: Supplementary file 1 [file molecules-25-05750-s001.pdf]

Supplementary data

Fe(III) complexes based on *mono-* and bis-pyrazolyl-*s*-triazine ligands; synthesis, molecular structure, Hirshfeld and antimicrobial evaluations.

Saied M. Soliman*, Hessa H. Al-Rasheed, Jörg H. Albering, Ayman El-Faham*

Table of Contents

**Method S1**	**Synthesis of 2,4-dihydrazino-6-substituted 1,3,5-triazine derivatives**
**Method S2**	**General method for the synthesis of dimethyl(1H-pyrazol-1-yl)-1,3,5-triazine derivatives**
**Method S3**	**Tested pathogenic microbes**
**Method S4**	**Agar well diffusion method**
**Method S5**	**Inhibition percentage of target microbe's growth at different concentrations of tested synthetic compounds**
**Method S6**	**Minimum inhibitory (MIC) and Minimum bactericidal concentration (MBC)**
**Figure S1**	**^{1}H NMR and ^{13}C NMR of ^{Morph}BPT**
**Figure S2**	**^{1}H NMR and ^{13}C NMR of ^{Pip}BPT**
**Figure S3**	**^{1}H- and ^{13}C-NMR for ^{bisMorph}PT**
**Figure S4**	**Structure showing the disordered parts in 2.**
**Figure S5**	**Hirshfeld surfaces mapped over d_{norm}, shape index and curvedness.**

Method S1: Synthesis of 2,4-dihydrazino-6-substituted 1,3,5-triazine derivatives

First, 2,4-dichloro-6-substituted 1,3,5-triazine derivatives were prepared following the reported method [1,2], then the dihydrazino derivatives was prepared following our reported method [2] and used directly into the next step.

***Method S2: General method for the synthesis of dimethyl(1H-pyrazol-1-yl)-1,3,5-triazine derivatives* [2]**

2,4-Dihydrazino-6-substituted-1,3,5-triazine (10 mmol) was dissolved in 20 mL DMF and then acetylacetone (25 mmol) was added, followed by addition of triethylamine (16 mmol) in 10 mL DMF with stirring at room temperature (in case of monohydrazino-triazine, 12mmol of aceylacetone and 8 mmol of triethylamine were used) . The reaction mixture was refluxed for 8 h, the progress of the reaction was monitored by TLC using ethylacetate-hexane (4:6). The solution was allowed to cool to room temperature, and then ice-cold water was added with continuous stirring. The reaction mixture was kept in an ice bath for 3 h, and the product was collected by filtration, washed with cold water (3 x 20 mL), and then dried under vacuum. The crude product was recrystallized from ethanol to afford the product.

*4-(4,6-*bis*(3,5-Dimethyl-1H-pyrazol-1-yl)-1,3,5-triazin-2-yl)morpholine (^Morph^**BPT**)*

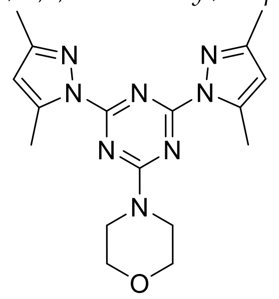

White crystals, mp = 187-188°C, in yield 82% (**B**); 70% (**A**); IR (KBr, cm^{-1}) 1664, 1620 (C=N), 1595 (C=N, C=C); ^{1}H NMR ($CDCl_3$): δ 2.27 (s, 6H, $2CH_3$),2.632 (s, 6H, $2CH_3$), 3.56 (t, 4H, *J* = 2.4 Hz, $2CH_2$), 3.86 (t, 4H, *J* = 4.4 Hz, $2CH_2$), 5.97 (s, 2H, 2CH) ppm; ^{13}C NMR ($CDCl_3$): δ 10.7, 12.9, 41.2, 63.4, 108.1, 140.5, 149.0, 160.5, 162.4 ppm.

*2,4-*bis*(3,5-Dimethyl-1H-pyrazol-1-yl)-6-(piperidin-1-yl)-1,3,5-triazine (^Pip^**BPT**)*

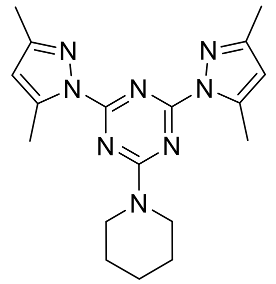

White crystals, mp = 141-142°C, in 96% yield (**B**); 73% (**A**); IR (KBr, cm^{-1}) 1674, 1624 (C=N), 1595 (C=N, C=C); ^{1}H NMR ($CDCl_3$): δ 1.60 (m, 6H, $3CH_2$), 2.27 (s, 6H, $2CH_3$),2.62(s, 6H, $2CH_3$), 3.78 (t, 4H, , *J* = 4.4 Hz, $2CH_2$), 5.97 (s,2H, 2CH) ppm; ^{13}C NMR ($CDCl_3$): δ 13.5, 15.8, 24.3, 25.5, 45.1, 110.8, 143.3, 151.7, 163.5, 164.7 ppm.

4,4′-(6-(3,5-Dimethyl-1H-pyrazol-1-yl)-1,3,5-triazine-2,4-diyl)dimorpholine (*^{bisMorph}**PT***)

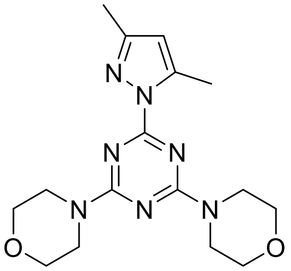

Off-white solid, mp 152-153 °C, yield 81%; IR (KBr, cm^{-1}): 1643, 1622 (C=N), 1553 (C=N, C=C); ^{1}H NMR ($CDCl_3$): δ 2.27 (s, 3H, CH_3), 2.59 (s, 3H, CH_3), 3.17 (t, 8H, *J* = 2.4 Hz, $4CH_2$), 3.82 (brs, 8H, $4CH_2$), 5.97 (s, 1H, CH) ppm; ^{13}C NMR ($CDCl_3$): δ 14.0 (CH_3), 16.0(CH_3), 43.6 (C-N-C, morpholine), 43.9 (C-N-C, morpholine), 66.7 (C-O-C, morpholine), 66.8 (C-O-C, morpholine), 110.4(C_b, pyrazole), 143.1(C_a, pyrazole), 151.3(C_c, pyrazole), 163.2(C=N, triazine),165.4(C=N, triazine) ppm.

Method S3: Tested pathogenic microbes

The antibacterial activity of ^{Pip}**BPT,** ^{Morph}**BPT** and ^{bisMorph}**PT** and their Fe(III) complexes were evaluated against Gram-positive bacteria namely; *Staphylococcus aureus* (ATCC 29213) and *Staphylococcus epidermidis ATCC 12228*; and Gram-negative bacteria namely; *Escherichia coli* (ATCC 25922) and *Pseudomonas aeruginosa* (*ATCC 27853*). Gentamycin was used as standard antibacterial agent. The samples maintained in Brain heart infusion (BHI) at 20°C; 300 mL of each stock–culture was added to 3 mL of BHI broth. Overnight cultures were kept for 24 h at 37 °C ± 1°C and the purity of cultures was checked after 24 h of incubation. After 24 h of incubation, bacterial suspension was diluted with sterile physiological solution, for the diffusion and indirect bioautographic tests, to 108 CFU/mL (turbidity = McFarland barium sulfate standard 0.5), in case of fungus *Candida albicans* (ATCC 60193), the used medium in antagonistic activity against tested fungi is Potato Dextrose Agar, where Fluconazole was used standard antifungal agent.

Method S4: Agar well diffusion method

Synthetic compounds were prepared at concentration 2 mg/mL dissolved in DMSO as stock solutions. Preparation of sterilized Mueller Hinton agar plates seeded with tested pathogenic bacteria occurred. The wells are done by sterilized cork borer in size 6 mm and hence 200 μg of the synthetic compound was poured in each well comparably with DMSO as control. The plates were incubated at 37°C for 24 h. after incubation period; antimicrobial activity was determined by inhibition zones.

Method S5: Inhibition percentage of target microbe's growth at different concentrations of tested synthetic compounds

The antibacterial activity of synthetic compounds was studied by employing a micro dilution method, using nutrient broth. The inoculum was prepared as described previously. Serial dilutions were performed in 96–well plate to reach concentrations ranging from 150 to 2.50 μg/mL, additionally as well as control (containing nutrient broth plus microbe, without antimicrobial substance and no DMSO) and blank samples (containing nutrient broth plus DMSO, without microbe and no antimicrobial substance). Each test and control well was inoculated with 5.0 μL of a microbial suspension (108 CFU/mL). Microplate reader measured the results at 630 nm wavelength.

Method S6: Minimum inhibitory (MIC) and Minimum bactericidal concentration (MBC)

Different dilutions of the compounds are inoculated with tested pathogenic microbes. After incubation period of 96 well microplate, the results are measured using microplate reader. To determine at what level the MIC and MBC endpoint is established; subculture of test samples at different concentrations occurred in nutrient agar plates.

Figure S1. ^{1}H NMR and ^{13}C NMR of ^{Morph}BPT

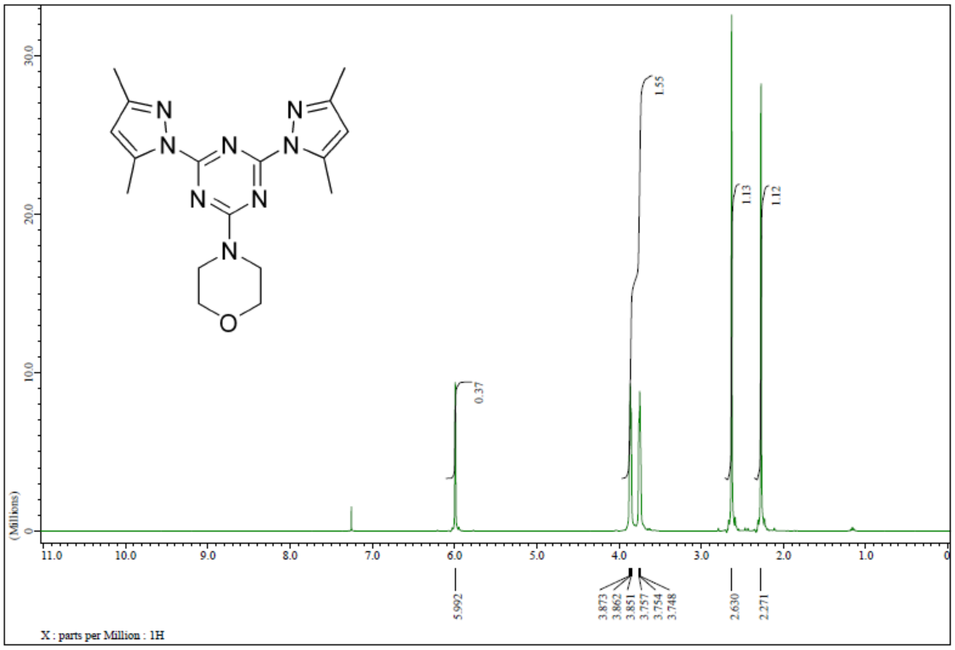

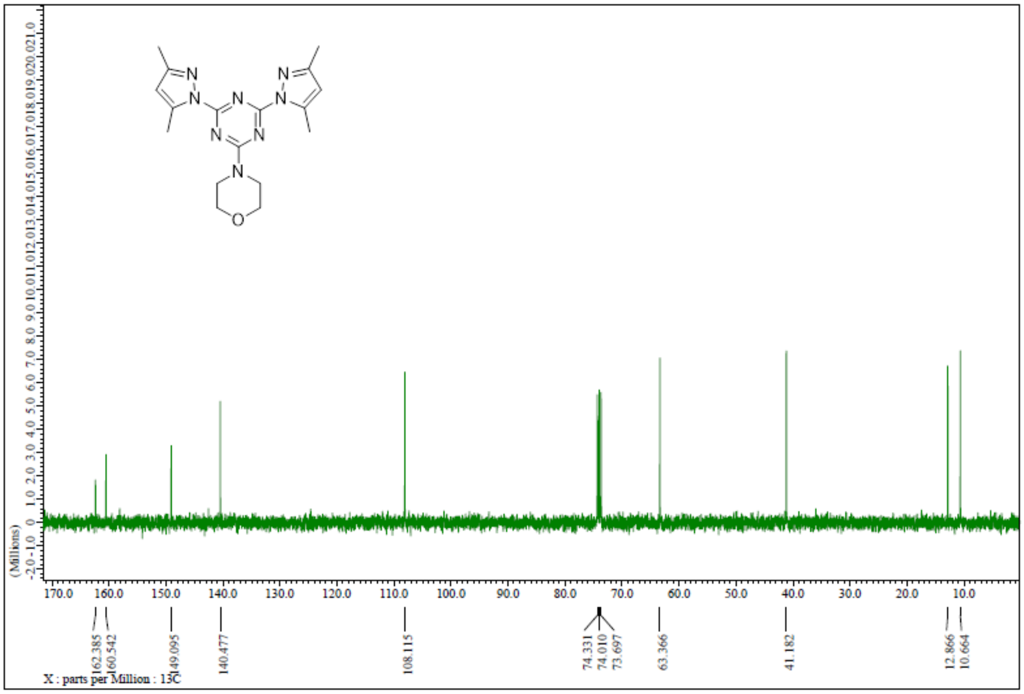

Figure S2. ^{1}H **NMR and** ^{13}C **NMR of** ^{Pip}**BPT**

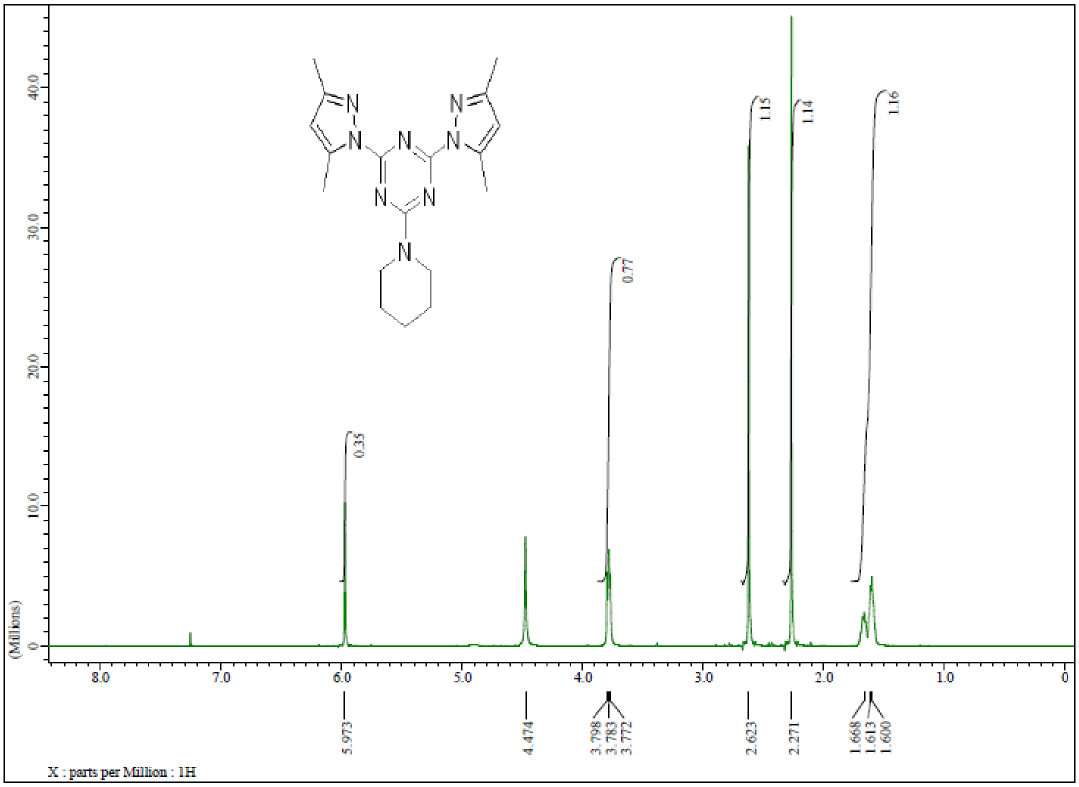

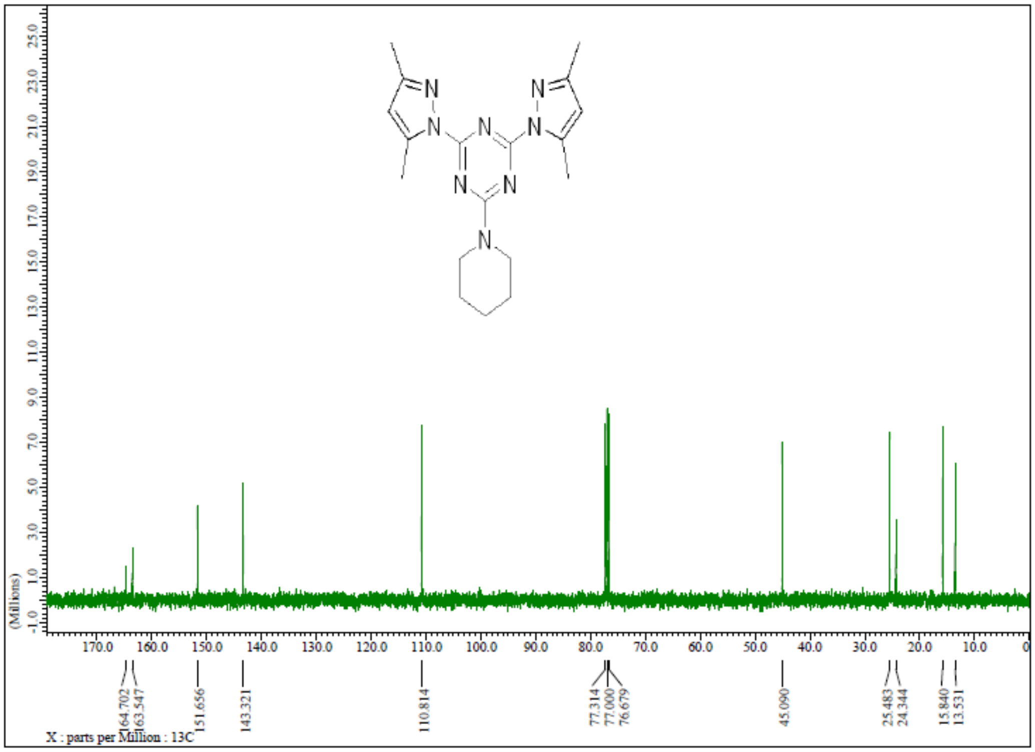

Figure S3. ^{1}H- and ^{13}C-NMR for ^{bisMorph}**PT**

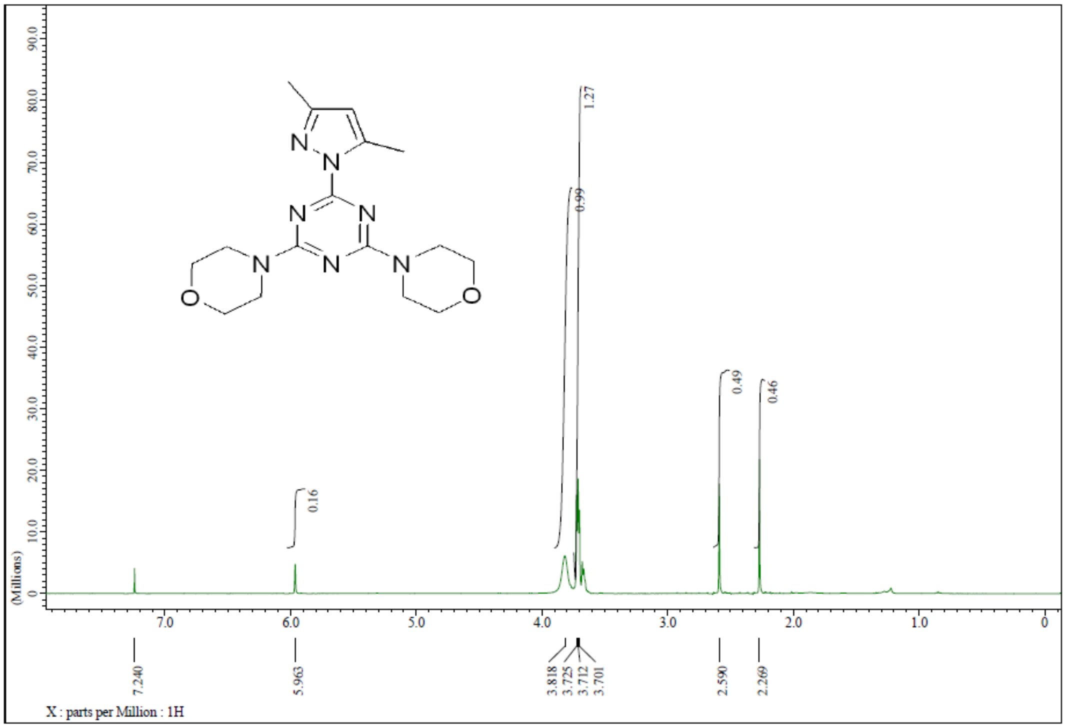

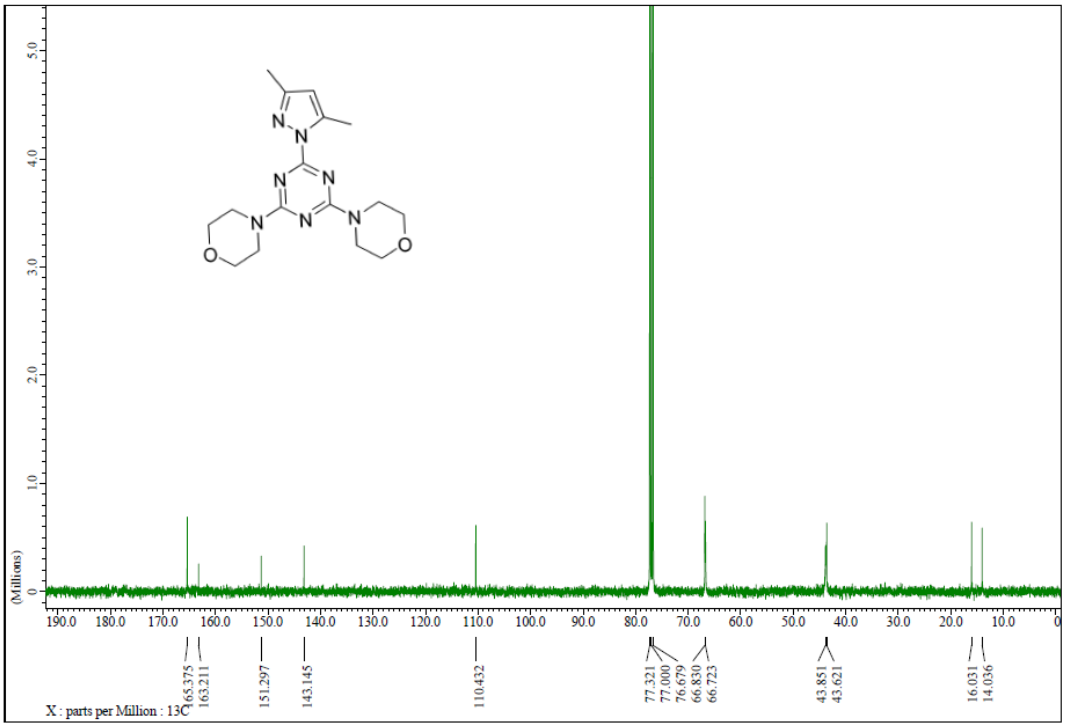

Figure S4 Structure showing the disordered parts in **2**.

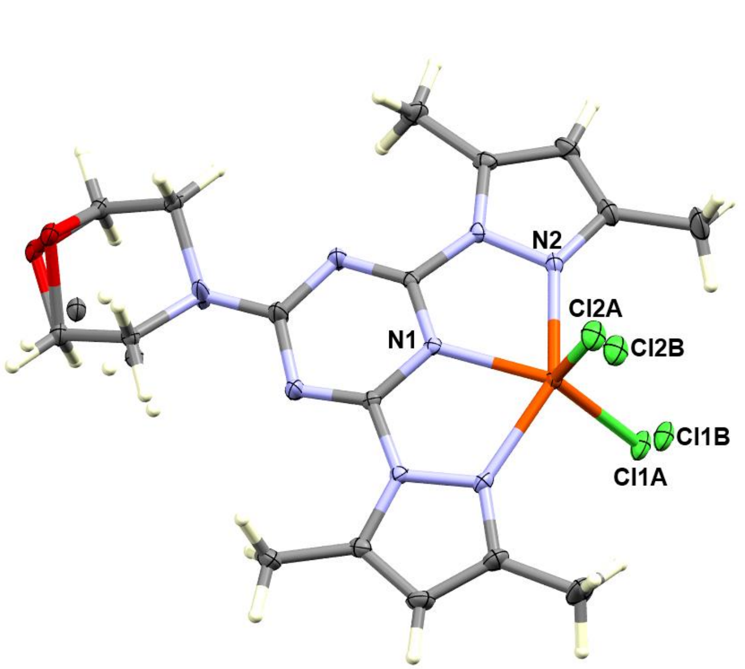

Figure S5 Hirshfeld surfaces mapped over d_{norm}, shape index and curvedness.

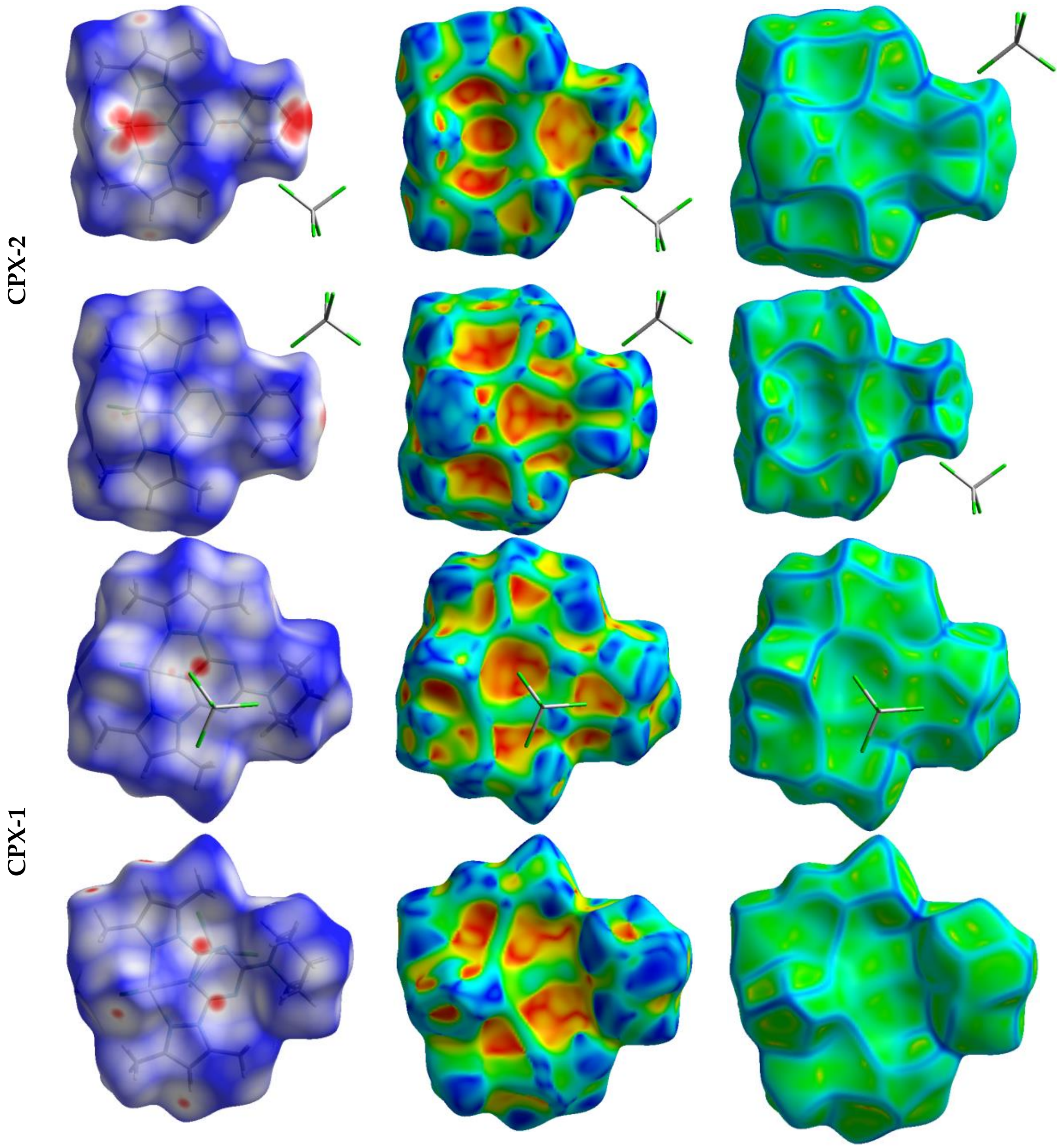

References

[1] A. El-Faham, K.A. Dahlous, Z.A. AL Othman, H.A. Al-Lohedan, G.A. El-Mahdy. Sym-trisubstituted 1, 3, 5-triazine derivatives as promising organic corrosion inhibitors for steel in acidic solution. *Molecules* , **2016**, 21, 436.

[2] A. El-Faham, S.M. Soliman, H.A. Ghabbour, Y.A. Elnakady, T.A. Mohaya, M.R. Siddiqui, F. Albericio. Ultrasonic promoted synthesis of novel s-triazine-schiff base derivatives; molecular structure, spectroscopic studies and their preliminary anti-proliferative activities. *J. Mol. Struc*. **2016**, 1125, 121-135.
